# Poly(3-hydroxybutyrate) Production from Lignocellulosic Wastes Using *Bacillus megaterium* ATCC 14581

**DOI:** 10.3390/polym15234488

**Published:** 2023-11-22

**Authors:** Lacrimioara Senila, Emese Gál, Eniko Kovacs, Oana Cadar, Monica Dan, Marin Senila, Cecilia Roman

**Affiliations:** 1 Research Institute for Analytical Instrumentation Subsidiary, National Institute for Research and Development of Optoelectronics Bucharest INOE 2000, 67 Donath Street, 400293 Cluj-Napoca, Romania; eniko.kovacs@icia.ro (E.K.); oana.cadar@icia.ro (O.C.); marin.senila@icia.ro (M.S.); cecilia.roman@icia.ro (C.R.); 2 Faculty of Chemistry and Chemical Engineering, Babes-Bolyai University, 11 Arany Janos Street, 400028 Cluj-Napoca, Romania; emese.gal@ubbcluj.ro; 3 Faculty of Horticulture, University of Agricultural Sciences and Veterinary Medicine, 3–5 Manastur Street, 400372 Cluj-Napoca, Romania; 4 National Institute for Research and Development of Isotopic and Molecular Technologies, 67–103 Donath Street, 400293 Cluj-Napoca, Romania; monica.dan@itim-cj.ro

**Keywords:** poly(3-hydroxybutyrate), microwave irradiation, lignocellulosic waste, *Bacillus megaterium* ATCC 14581

## Abstract

This study aimed to analyze the production of poly(3-hydroxybutyrate) (PHB) from lignocellulosic biomass through a series of steps, including microwave irradiation, ammonia delignification, enzymatic hydrolysis, and fermentation, using the *Bacillus megaterium* ATCC 14581 strain. The lignocellulosic biomass was first pretreated using microwave irradiation at different temperatures (180, 200, and 220 °C) for 10, 20, and 30 min. The optimal pretreatment conditions were determined using the central composite design (CCD) and the response surface methodology (RSM). In the second step, the pretreated biomass was subjected to ammonia delignification, followed by enzymatic hydrolysis. The yield obtained for the pretreated and enzymatically hydrolyzed biomass was lower (70.2%) compared to the pretreated, delignified, and enzymatically hydrolyzed biomass (91.4%). These hydrolysates were used as carbon substrates for the synthesis of PHB using *Bacillus megaterium* ATCC 14581 in batch cultures. Various analytical methods were employed, namely nuclear magnetic resonance (^1^H-NMR and^13^C-NMR), electrospray ionization mass spectrometry (EI-MS), X-ray diffraction (XRD), Fourier transform infrared spectroscopy (FT-IR), and thermogravimetric analysis (TGA), to identify and characterize the extracted PHB. The XRD analysis confirmed the partially crystalline nature of PHB.

## 1. Introduction

Synthetic and non-biodegradable plastics derived from fossil fuels are widely employed in multiple sectors, including the packaging industry, technology, and medicine, as well as everyday life. The extensive consumption of plastic has resulted in inefficient disposal practices, contributing to environmental pollution. In this regard, serious concerns exist regarding environmental pollution that should encourage the use of renewable resources for the production of fuels and plastics [1,2].

Polyhydroxyalkanoates (PHAs) are aliphatic polyesters with structures based on 3-hydroxycarboxylic acid. Due to their chemical compositions and physical properties, PHAs are defined as ecological, biocompatible, nontoxic, and thermostable plastics; therefore, they are a viable alternative to traditional synthetic plastics [3,4,5]. The physicochemical characteristics of PHAs are determined based on the polyester chain’s composition, the organism that produces it, the culture conditions, and the extraction method used. Based on the length of the polymeric chain, there are three types of PHAs: short-chain-length PHAs (scl) with 3–5 carbon atoms, medium-chain-length (mcl) PHAs with 6–14 carbon atoms, and long-chain-length (lcl) PHAs with more than 15 carbon atoms [6,7,8,9,10].

Polyhydroxyalkanoates are thermoplastic polyesters with different structures such as poly(3-hydroxybutyrate) (PHB), polyhydroxy valerate (PHV), poly-hydroxybutyrate-*co*-hydroxyvalerate (PHBV), etc. [9]. PHAs consisting of different monomers have a specific melting points, hydrophobicities, and degrees of crystallinity. scl-PHAs species have melting points greater than 170 °C and crystallinities that exceed 60%, whereas mcl-PHA compounds have lower crystallinities (<40%), lower melting points, and high elasticities. Yan et al. (2021) produced PHAs (620.7 mg COD/L) from lignocellulosic biomass (i.e., various rubber wood types, sugarcane bagasse, sorghum stalk, cassava stalk) after pressurized hot water pretreatment, enzymatic hydrolysis, and the anaerobic fermentation of sugars with activated sludge [11]. The microbial strains used for the production of PHAs are as follows: *Ralstonia eutropha*, *Pseudomonas oleovorans*, *Azotobacter vinelandii*, *Actinobacillus* sp., *Alcaligenes latus* and *Bacillus* sp., *Burkholderia sacchari*, and *Halomonas* spp. [12,13]. The unique feature of these polymers compared to other polymer types is their occurrence inside of living organisms [14]. 

Gram-negative bacteria, mainly *Protomonas*, *Pseudomonas*, *Vibrio*, *Xanthomonas*, *Alcaligenes*, *Azotobacter*, *Burkholderia*, *Citrobacter*, *Cupriavidus*, *Enterobacter*, *Klebsiella*, and *Methylobacterium*; Gram-positive bacteria, mainly Bacillus, *Corynebacterium*, *Streptomyces*, *Rhodococcus*, *Nocardia*, and *Staphylococcus*; cyanobacteria genera, mainly *Spirulina*, *Synechocystis*, *Nostoc*, and *Aulosira*; and haloarchaea bacteria, mainly *Halomonas*, *Halogranum*, *Haloferax*, *Haloterringena*, and *Halopiger*, are used for PHB production. Khomlaem et al. (2023) documented the production of PHAs and astaxanthin from lignocellulosic biomass using cultures of two strains: *Bacillus megaterium* ALA2 and *Paracoccus* sp. LL1. A concentration of 101.7 g/L PHAs was reported in this study [15]. The production of PHAs from lignocellulosic biomass requires various steps, such as pretreatment, enzymatic hydrolysis, and fermentation with microbial strains. Cellulose, the first component of lignocellulosic material, can be separated, and the monomer sugars can be fermented to form PHAs. Since the carbon source is the main factor that affects the production of PHAs, the isolation of carbon sources from wastes represents a solution to reduce the cost of PHA production. Several carbon sources have been used as substrates for PHAs production, including fructose, glucose, lactic acid, xylose, propionic acid, and different lignocellulosic biomasses [16].

The available strategies of biomass waste pretreatment are classified as physical, chemical, physical–chemical, and biological. Recently, acetic acid was used as a carbon source for poly-*β*-hydroxybutyrate using *Bacillus cereus* L17 strain [17]. In some studies, *Halomonas* sp., a Gram-negative bacterium belonging to the Halophile family that can tolerate a saline medium, was used for the production of PHB from glycerol and glucose [18]. This bacterial strain can be isolated from hypersaline meromictic waters, like those of Fără Fund Lake [19]. 

*Bacillus megaterium* is a Gram-positive bacterium with the ability to use glucose, galactose, fructose, xylose, maltose, lactose, and sucrose as its sole carbon sources for growth. [20]. In the past, *Bacillus megaterium* was used for the production of vitamins and proteins [21]. Nevertheless, the production of poly(3-hydroxybutyrate-*co*-4-hydroxybutryate) copolyester has also been reported [22]. Israni et al. (2020) used *Bacillus megaterium* sp. Ti3 for the production of PHAs from cheddar cheese whey and reported a productivity of 0.05 g/L/h [22]. In a previous study, poly(lactic acid) (PLA) production from renewable lignocellulosic wastes was documented [23]. In this context, the current study seeks to obtain another biodegradable polymer, using *Bacillus megaterium* as a strain for microbial fermentation. The aim of this study was to investigate (a) the microwave pretreatment of fruit cutting wastes, (b) ammonia delignification, (c) the enzymatic hydrolysis of pretreated biomass with complex enzymes, (d) the fermentation of sugars with *Bacillus megaterium* in order to obtain PHB, (e) purification, (f) the and chemical and structural characterization of the obtained PHB.

## 2. Materials and Methods

### 2.1. Reagents and Strains

All the chemicals used in the methodology were of analytical grade. Sodium chlorite (80%) (NaClO_2_) was purchased from Alfa Aesar GmbH and Co. (Karlsruhe, Germany). Ammonia, 3.5-dinitrisalycilic acid (DNS), sodium hydroxide (NaOH), 3-hydroxybytyric acid (C_4_H_8_O_3_), 3-hydroxypentanoic acid (C_5_H_10_O_3_), dipotassium phosphate ((K_2_HPO_4_), D(+)-glucose (C_6_H_12_O_6_), magnesium sulphate (MgSO_4_), calcium chloride (CaCl_2_), ammonium sulphate ((NH_4_)_2_SO_4_), sodium phosphate dibasic dodecahydrate (Na_2_HPO_4_.12H_2_O), ammonium ferric citrate (NH_4_)_5_[Fe(C_6_H_4_O_7_)_2_], boric acid (H_3_BO_3_), cobalt(II) chloride hexahydrate (CoCl_2_.6H_2_O), zinc sulfate heptahydrate (ZnSO_4_.7H_2_O), sodium molybdate dihydrate (NaMoO_4_.2H_2_O), nickel(II) chloride hexahydrate (NiCl_2_.6H_2_O), copper(II) sulfate pentahydrate (CuSO_4_.5H_2_O), magnesium chloride hexahydrate (MgCl_2_.6H_2_O), sodium azide (NaN_3_), meat extract, peptone, sulfuric acid (H_2_SO_4_), and chloroform (CHCl_3_) were purchased from Merck (Darmstadt, Germany). *Trichoderma reesei* ATCC 26921 and *β*-glucosidase derived from almonds used for hydrolysis were purchased from Sigma-Aldrich (St. Louis, MO, USA). *Bacillus megaterium* ATCC 14581 was purchased from Microbiologics (Cooper Avenue North, St. Cloud, MN, USA). All the solutions were prepared using ultrapure water (18.2 MΩcm^−1^ at 20 °C) obtained using a Direct-Q3 UV Water Purification System (Millipore, Molsheim, France). The cherry orchard biomass was purchased from the “Ion Ionescu de la Brad” University of Life Sciences of Iasi, Romania. 

### 2.2. Microwave Irradiation of Biomass Using Response Surface Methodology (RSM)

The pretreatment method’s experimental design involved the application of a central composite design (CCD), which assessed the interplay between dependent and independent variables through RSM. Both RSM and CCD were applied in this study to determine the optimal conditions for the high recovery of cellulose and solid yield from the lignocellulosic biomass. The Minitab software 17.0 (Minitab, LLC, State College, PA, USA) was used. Four response variables, namely solid yield, cellulose, hemicellulose, and lignin, with three levels, namely high (+1), midpoint (0), and low (−1), were used in combination, and they are shown in Table 1. The quadratic model and regression equations were applied according to the method of Gunalan et al. (2023) [24]. 

RSM was used for the analysis of variance for each dependent variable. The linear, square, and two-way interaction models were applied for the interactions between input variable. For each dependent variable, a 3D surface plot was generated that illustrated its relationship with the independent variables. Additionally, principal component analysis was performed to construct a correlation matrix. Each experiment was carried out in triplicate and standardized to obtain the maximum cellulose component. The pretreatment components’ experimental values were compared to the values predicted using the model [25]. 

### 2.3. Delignification of the Pretreated Biomass with Ammonia

The pretreated lignocellulosic biomass was reacted with 20% (*w*/*v*) ammonia for 12 h at 80 °C according to the method of Thuoc et al. (2021), albeit with modifications [26]. The solid fraction was dried at 105 °C and used for the enzymatic hydrolysis step. The chemical composition of the delignified biomass was analyzed to determine its components.

### 2.4. Enzymatic Hydrolysis of the Delignified Biomass 

The enzymatic hydrolysis of the pretreated and delignified biomass was carried out in a 250 mL Erlenmeyer flask loaded with 0.05 M citrate buffer (pH 4.8). In order to prevent contamination, sodium azide (5 mg/mL) was added to the mixture, and cellulase enzymes were used for enzymatic hydrolysis. The concentration of cellulase was 25 FPU/g of substrate in all the tests. The temperature was set at 50 °C for 24, 48, and 72 h. The reducing sugars content were analyzed using the Miller method [27]. After saccharification, the mixtures were heated at 80 °C in order to deactivate the enzyme and concentrate the solution used for further PHB production. 

### 2.5. PHA Production via Fermentation with Bacillus Megaterium ATCC 14581

#### 2.5.1. Preparation of Stock Culture and Cultivation Conditions 

The *Bacillus megaterium* ATCC 14581 lyophilized culture was purchased from Microbiologics (Cooper Avenue North, St. Cloud, MN, USA). For reactivation and routine growth, a liquid nutrient broth (NB) containing (g/L) 3.0 meat extract and 5.0 peptone was used. For PHB production, a broth medium was used that had the following composition (g/L): 10 g glucose, 1.5 g KH_2_PO_4_, 4.45 g Na_2_HPO_4_.12H_2_O, 0.2 g MgSO_4_, 0.01 g CaCl_2_, 1 g(NH_4_)_2_SO_4_, 5 g Fe(III)-NH_4_ citrate, 1 g yeast extract, and trace elements (300 mg H_3_BO_3_, 200 mg CoCl_2_.6H_2_O, 100 mg ZnSO_4_.7H_2_O, 30 mg NaMoO_4_. 2H_2_O, 20 mg NiCl_2_.6 H_2_O, 10 mg CuSO_4_.5H_2_O, and 30 mg MgCl_2_.6H_2_O). The strain was grown in flasks in NB for 8 h at 35 °C and centrifuged at 250 rpm. The medium was selected according to the method of Dietrich et al. (2020) [28]. The pH was maintained at 6.8 with 2N H_2_SO_4_ or 5% NaOH (*w*/*v*). 

#### 2.5.2. PHB Production from Lignocellulosic Biomass 

The hydrolysates obtained after the enzymatic hydrolysis of the pretreated biomass were subjected to fermentation with *Bacillus megaterium* bacteria. The experiments were performed in a 1.7 L bioreactor (Lambda Minifor, Lambda Laboratory Instruments, Brno, Czech Republic). The experiments were conducted at 35 °C for 48 h. The volume of the inoculum in the bioreactor was 10% (*v*/*v*). 

#### 2.5.3. PHB Isolation and Purification 

The PHB extraction was carried out according to the protocol previously described by Kucera et al. (2018) [29] and Hathi et al. (2022) [30]. After the fermentation, the culture was centrifuged at 5000 rpm for 30 min. The obtained pellets were resuspended in 10 mL of chloroform and heated at 100 °C for 1 h. The solvent was evaporated, and the polyester was precipitated with cold methanol. The polyester was filtered and purified through dissolution in chloroform and hexane (1:1, *v*/*v*). The PHB productivity rate was calculated by dividing the final PHB quantity (g/L) by the fermentation period (48 h). The PHB yield coefficient obtained using the cell biomass was calculated by dividing the final PHB quantity (g/L) by the dry cell weight (g/L). The dry cell weight (DCW) was quantified by weighting the cells after centrifugation and drying.

### 2.6. Chemical Characterization 

#### 2.6.1. Chemical Characterization of the Raw and Pretreated Biomass

The cellulose, hemicellulose, and lignin contents from the raw and the pretreated and delignified biomass were determined according to the method of Senila et al. (2020) [31]. The total nitrogen (N), carbon (C), hydrogen(H), and sulfur (S) contents were determined via combustion using a Flash EA 2000 CHNS/O analyzer (Thermo Fisher Scientific, Waltham, MA, USA). The samples’ moisture contents were determined after drying in a universal oven (UFE 400, Memmert, Germany) at 105 °C for 24 h until constant mass. The ash contents were determined after the samples were calcinated at 550 °C.

#### 2.6.2. Reducing Sugar Analysis

The reducing sugar analysis was performed according to the dinitrosalicylic acid (DNS) method [27], including the reaction of glucose with 3,5-dinitrosalycilic acid, the formation of gluconic acid and 3-amino-5-nitrosalycilic acid, and the variation in the color from yellow to intense red.

#### 2.6.3. PHB Analysis via Gas Chromatography (GC)

The analysis of PHB was carried out via gas chromatography coupled with a flame ionization detector (FID, Agilent Technologies 7683, Santa Clara, CA, USA). The method was developed based on the method of Bhati et al. (2010), albeit with modifications [32]. Methyl esters of 3-hydroxybytyric acid and 3-hydroxypentanoic acid standards were injected into GC-FID. The ester contents were determined using GC-FID (Agilent Technologies 6890 N, Santa Clara, CA, USA) equipped with a Zebron ZB-WAX capillary column (30 m × 0.25 mm × 0.25 µm). Helium was used as a carrier gas with a constant flow rate of 1 mL/min. The initial oven temperature was set to 50 °C for 1 min, before being increased to 200 °C at a rate of 10 °C min for 2 min, and, finally, the temperature was increased to 220 °C at a rate of 5 °C min^−1^ for 20 min. The FID and injector temperatures were set at 250 °C.

### 2.7. Structural Characterization of the Obtained PHB 

#### 2.7.1. Electrospray Ionization High Resolution Mass Spectrometry (ESI-HRMS), Proton Nuclear Magnetic Resonance (^1^H-NMR), Carbon Nuclear Magnetic Resonance (^13^C-NMR) 

Proton nuclear magnetic resonance (1H NMR) and carbon nuclear magnetic resonance (13C NMR) spectra were recorded at room temperature using a Bruker Advance spectrometer (Billerica, MA, USA) in CDCl_3_. The high-resolution mass spectrometer experiment was carried out using an ion-trap tandem mass spectrometer (Thermo Scientific LTQ Orbitrap XL, Waltham, MA, USA) equipped with an ESI source. The ESI-HRMS spectra were acquired in a positive mode.

#### 2.7.2. TGA/DTG Analysis

The thermal behavior of the PHB was investigated using a TA Instrument SDT O 600 (TA Instruments, New Castle, DE, USA) under air atmosphere from 30 °C to 1000 °C and at a heating rate of 10 °C/min. The weight loss was determined from the curve (DTG). 

#### 2.7.3. X-ray Diffraction (XRD)

The X-ray diffraction pattern was conducted using a Bruker D8 Advance diffractometer with CuKα anode (λ = 1.54060 Å) in the range 2θ = 10–40°. A silicon zero-background plate was used to ensure that there was no peak related to the sample holder. The crystallinity degree was calculated as the ratio of the total area under the crystalline peaks to the total area under the crystalline and amorphous area [33]. 

#### 2.7.4. FT-IR Spectroscopy

The FT-IR spectra of the PHB were obtained using a Bruker Vector 22 FT-IR spectrometer (Bruker, USA) in the range of 600 to 4000 cm^−1^. The samples were prepared by mixing the sample with KBr at a 1:100 ratio. The pellets were kept in vacuum desiccators.

## 3. Results

### 3.1. Chemical Compositions of the Raw Biomass

The elemental analysis of the raw biomass showed the following results: 1.01% N, 45.7% C, 5.75% H, and 0.18% S. The content of ash was 4.86%. Biomass contains 40.0% cellulose, 21.95% hemicellulose, and 29.62% lignin. The content of 62% holocellulose is shown to be a great source of sugars that can be extracted and transformed via selective fermentation into PHB. The moisture content was 6.86%.

### 3.2. Chemical Compositions of the Pretreated Biomass Using Response Surface Methodology

During the microwave irradiation procedure, the biomass changed from white to yellow, before changing to brown; the color intensity increases along with the temperature and the reaction time. The solid yield and chemical composition of the pretreated biomass are presented in Table 2. 

The solid yield decreased with the increase in the pretreatment temperature and the reaction time from 75.2% to 42.3%. Lower temperature and microwave power had a positive influence on the yields. At high temperatures, the hemicelluloses break down, and the cellulose partially dissolves, leading to thermal degradation and a reduction in the solid yield [34]. The depolymerization of hemicelluloses and the relocation of crystalline cellulose are caused by hydronium ions from water during microwave pretreatment at high temperatures. The solid phase resulting from the pretreatment was analyzed to determine the cellulose, hemicellulose, and lignin contents.

**Figure 1:**
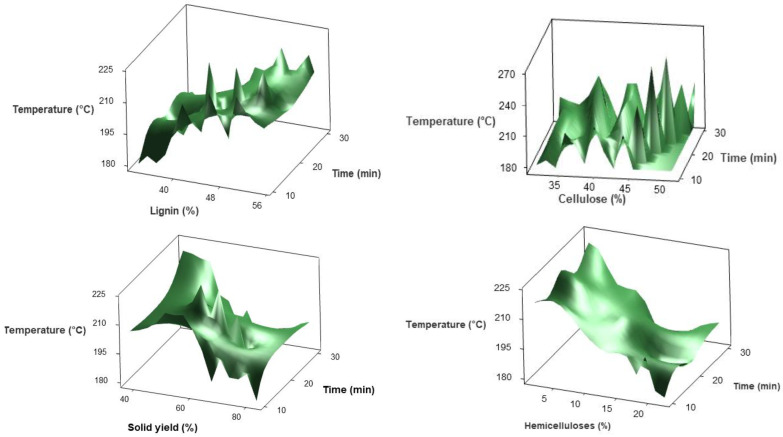
Three-dimensional response surface plots of the effect of temperature and time content on the cellulose yield.

The Minitab software was used to fit the obtained data via a multiple regression analysis of the studied variables’ interaction. The results were presented using contour plots and surface plots. Two factors (temperature and time) were used for the central composite design. The model summary is presented in Table 3. The model fits the experimental data with a high correlation coefficient. The analysis of variance for the solid yield is presented in Table 4, and the analysis of variance for cellulose, lignin, and hemicellulose is presented in Supplementary Tables S1–S3. Due to its solubilization in the liquid fraction, the content of hemicellulose decreases upon increasing the temperature and reaction time. According to the high R^2^ values (95.95%, 91.29%, 90.68%, and 91.25%, respectively), the accuracy of the RSM model was accepted. The 3D response surface plots of the effects of cellulose on temperature and time content are presented in Figure 1. The optimal solution predicted using the model for the high cellulose content in the solid fraction is a temperature of 190.16 °C and a reaction time of 30 min.

The model was optimized to achieve the maximum cellulose content. The data were fitted to linear, interactive square, and two-way interaction methods. The accuracy of the model, the sum of squares, and the lack of fit were determined. The effects of temperature and time on cellulose recovery were analyzed. The temperature and reaction rate have implications for the solid yield, whereas time has no significant influence on the pretreated composition. A higher pretreatment temperature causes increased cellulose and lignin contents and a decreased hemicellulose content. Temperature is a critical parameter involved in controlling the microwave process of biomass. The model is statistically significant if the *p*-value is less than 0.05. The linear model’s *p*-value for solid yield and hemicellulose is <0.0001. The *p*-value of the square and two-way interaction for cellulose, hemicellulose, and lignin contents are significant for the temperature and insignificant for the reaction time.

The regression equations for dependent variables (Equations (1)–(5)) are presented below:

Solid yield (%) = −49 + 1.45 X_1_ + 1.39 X_2_ − 0.00428 X_1_^2^ − 0.0213 X_2_^2^ − 0.000612 X_1_X_2_ (1)

Cellulose (%) = 610 − 5.82X_1_ + 0.16 X_2_ + 0.01467 X_1_^2^ + 0.0002 X_2_^2^ + 0.00013 X_1_X_2_ (2)

Hemicellulose (%) = −43.6 + 1.093 X_1_ − 1.447 X_2_ − 0.00387 X_1_^2^ + 0.00751 X_2_^2^ + 0.00424 X_1_X_2_ (3)

Lignin (%) = −451 + 4.76X_1_ + 0.01 X_2_ − 0.01146 X_1_^2^ − 0.0003 X_2_^2^ +0.001 X_1_X_2_ (4)

Solid compositions (%) = 66 +0.61 X_1_ − 2.60 X_2_ − 0.0022 X_1_^2^ + 0.0234 X_2_^2^ + 0.00904 X_1_X_2_ (5)

**Figure 2:**
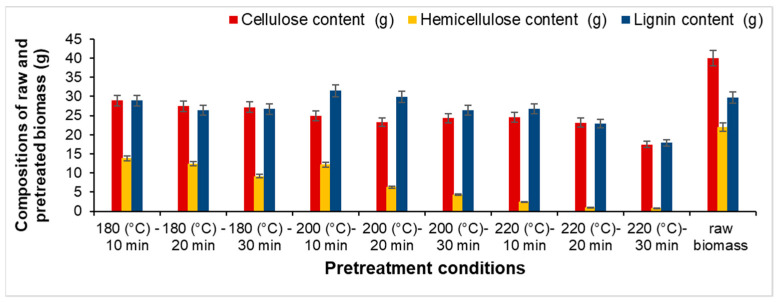
Compositions of the raw and pretreated biomass.

The compositions of the raw and pretreated biomass can be seen in Figure 2.

### 3.3. Composition of Delignified Biomass 

In this study, ammonia pretreatment was applied in order to improve the sugar yield for PHB production. According to Sipponen et al. (2019), the aqueous ammonia pre-treatment of biomass favors the isolation of lignin and facilitates the enzymatic hydrolysis of the cellulosic fraction [35].

**Figure 3:**
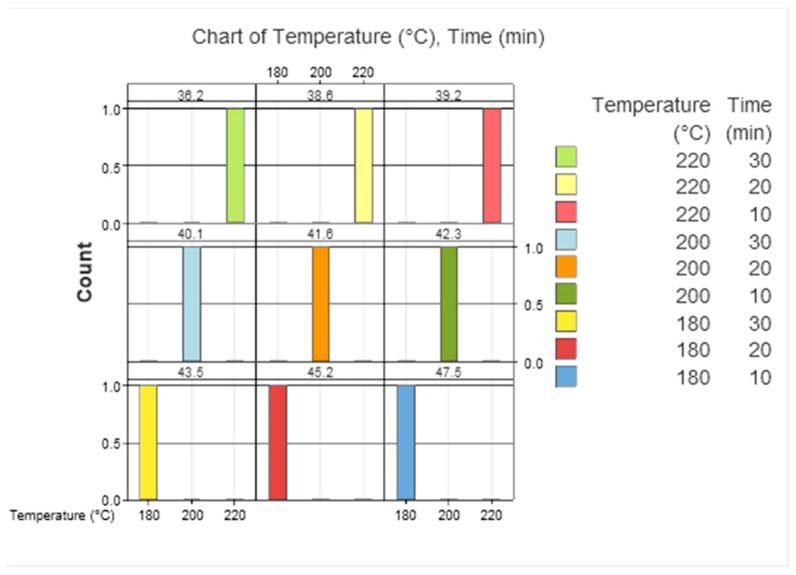
Solid yields obtained after delignification based on the temperature and reaction time.

**Figure 4:**
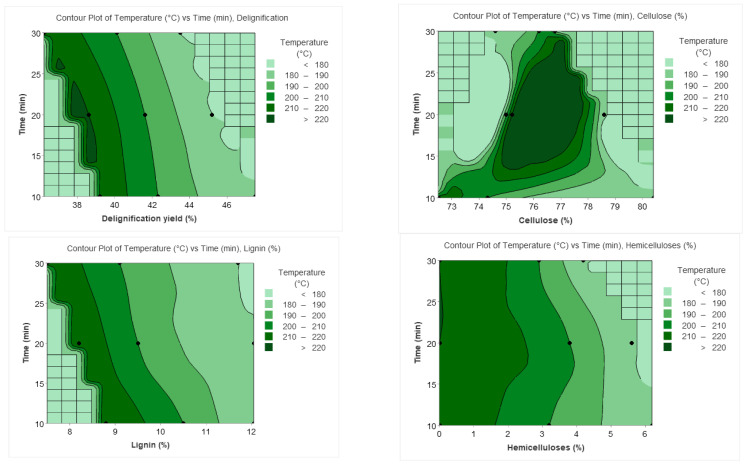
Contour plots of solid yields obtained after the delignification and chemical composition of residual solids based on the temperature and reaction time.

The results show that the solid yields (47.5–36.2 g/100 g delignified biomass) decrease as the pretreatment temperature increases due to the solubilization of the lignin fraction in the liquid phase (Figure 3). The delignification method eliminates almost 75% of the lignin content. The lignin content decreases (12.05–7.5%) with the increase in temperature (Figure 4). At 200 °C, all the hemicelluloses were eliminated. The cellulose content of the delignified biomass (80.4%) is higher than that of the raw biomass. The dissolution of lignin takes place via the removal of an acetyl group from the lignin structure. Pretreatment with 20% ammonia led to a loss of almost 40% of the biomass, and our results are in agreement with those of Tsafrakidou et al. (2023) [36]. Also, Park and Kim (2012) [37] reported rice straw and barley pretreatment via soaking in aqueous ammonia.

### 3.4. Enzymatic Hydrolysis of Pretreated and Delignified Biomass

**Figure 5:**
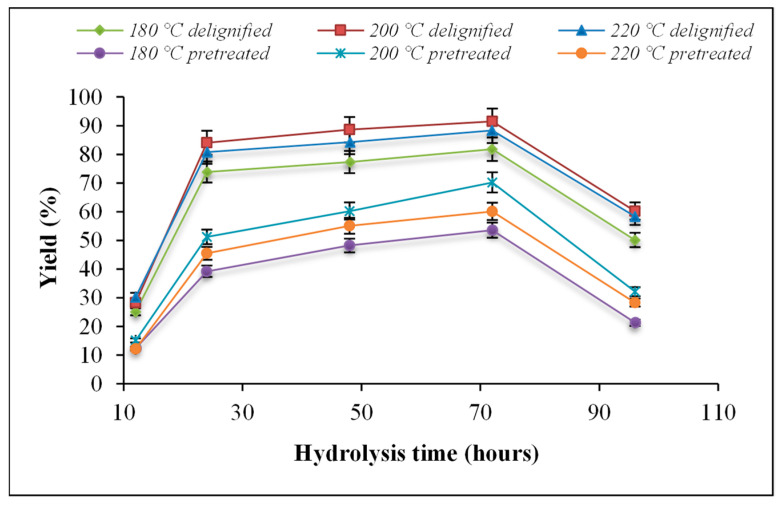
Enzymatic hydrolysis yields.

The cherry orchard biomass was treated with *Trichoderma reesei* ATCC 26921 and *β*-glucosidase enzymes. Figure 5 presents the yield of the obtained reducing sugars during the 24, 48, 72, and 96 h hydrolysis time points. The biomass was used for sugar solubilization in the presence of cellulase enzymes. The hydrolysis yields increased substantially when a delignified substrate was used for enzymatic hydrolysis (Figure 5). This indicates that the substrate compositions affect the hydrolysis process [38]. The presence of lignin in hydrolysate acts as an inhibitor of the enzymatic hydrolysis process. The pretreated (at 200 °C) and delignified biomass provided the highest hydrolysis yield (91.4%). After a hydrolysis period of 72 h, the pretreated biomass could reach a peak in the enzymatic hydrolysis yield of 70.2%. In all the experiments conducted, the prolonged enzymatic hydrolysis decreased the enzymatic hydrolysis yield, which was explained by the formation of inhibitory compounds. 

### 3.5. PHB Production by Bacillus Megaterium ATCC 14581

The production of PHB from lignocellulosic wastes as a renewable source has the advantage of decreasing the final production cost. However, sterilizing the culture medium is necessary [39]. The hydrolysates obtained after the enzymatic hydrolysis of the solid fraction, resulting from microwave irradiation pretreatment, were treated with *Bacillus megaterium*. The source of carbon determines the yield of the PHB obtained. According to Yamane (1992), if the carbohydrates are used as a carbon source, the yield of PHB is 86/180 = 0.478 [40]. Fermentation was carried out using the hydrolysates generated from the hydrolysis of delignified biomass, which contains glucose as its main component. The initial hydrolysate concentration was 10 g/L. During the enzymatic hydrolysis, some degraded substances, like hydroxymethyl furfural, acetic acid, and lignin degradation product, are formed, which further effects the fermentation process. *Bacillus megaterium* ATCC 14581 does not have the ability to ferment pentoses to create PHB, although it can produce PHB in the presence of fermentation inhibitors. In the first 10–30 h, the sugar consumption remains constant, then it rapidly increases due to PHB formation. The maximum DCW was 4.2 g/L, the PHB accumulation was 72%, PHB titer was 2.7 g/L, and the PHB productivity was 0.063 g PHB/L/h. After 48 h of fermentation, the PHB concentration was 3.024 g/L. The residual biomass was 1.176 g/L. The maximum PHB formation was obtained after 48 h of fermentation. 

From 100 ± 1.5 g of lignocellulosic biomass, 62.1 ± 3.1 g of the solid phase was separated after microwave irradiation. After ammonia delignification, 25.8 ± 1.8 g of the solid part was recovered. The enzymatic hydrolysis process produced 21.3 ± 1.1 g of glucose, which was then fermented with *Bacillus megaterium* ATCC 14581 to generate 7.3 ± 1.1 g of PHB. The fermentation yield was 72%. 

The results obtained and the previous literature data are presented in Table 5. 

According to Schmid et al. (2019) [46], bacteria from the Bacillus genera have the ability to form endospore, which affects the fermentation process.This study demonstrates that Bacillus megaterium ATCC 14581 can successfully produce PHB from the hydrolysates obtained from the lignocellulosic biomass. 

### 3.6. Structural and Termal Characterization of PHB

**Scheme 1:**
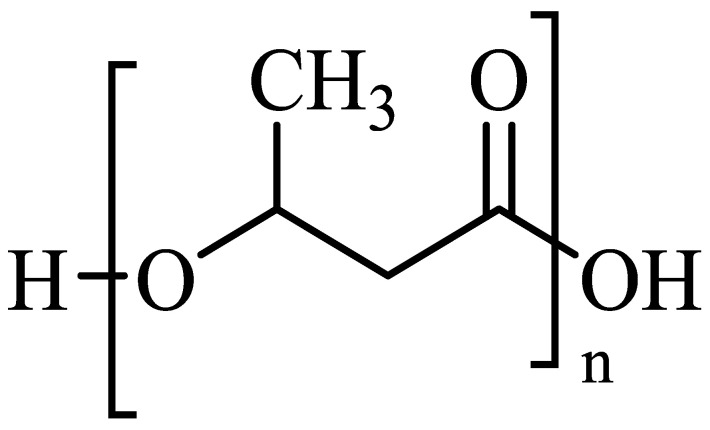
Chemical structure of PHB.

The chemical structure of the PHB is presented in Scheme 1.

#### 3.6.1. Proton Nuclear Magnetic Resonance (^1^H-NMR) 

**Figure 6:**
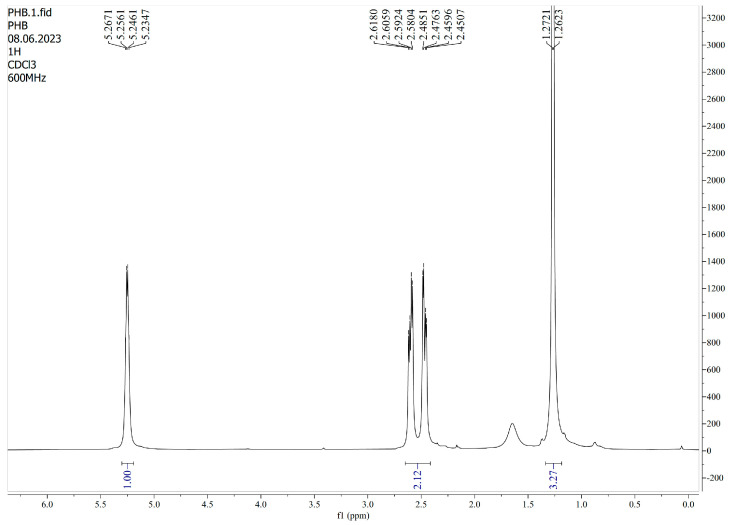
The ^1^H-NMR spectrum of PHB produced by *Bacillus megaterium* ATCC 14581 using lignocellulosic biomass as an additional carbon source in the medium.

^1^H-NMR is a remarkable procedure used for confirming the chemical structure of PHB. [47]. The ^1^H-NMR of the obtained PHB can be seen in Figure 6. In accordance with the literature, three signals can be observed: at 1.27 ppm (doublet, CH_3_), at 2.48–2.58 ppm (double quadruplet, CH_2_), and at 5.25 ppm (multiplet, CH), respectively. The obtained results are consistent with the ^1^H-NMR reported for the PHB produced by *Bacillus* spp. [48] and the PHB produced via the fermentation of fructose using *Cupriavidus necator* [16]. 

#### 3.6.2. ESI-HRMS Spectra

**Figure 7:**
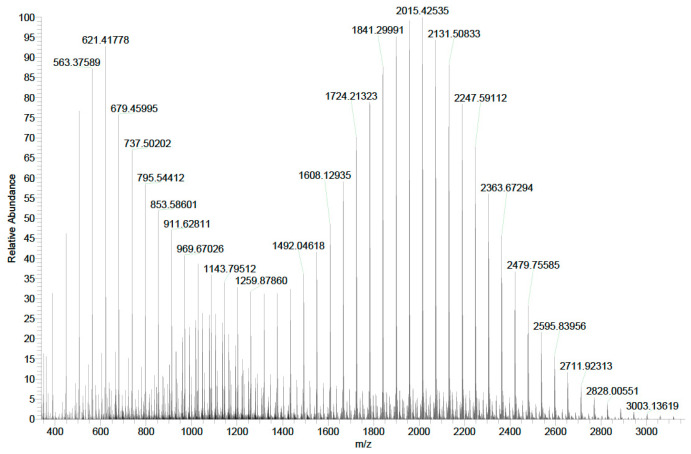
ESI (+)-HRMS spectrum of sample PHB.

**Figure 8:**
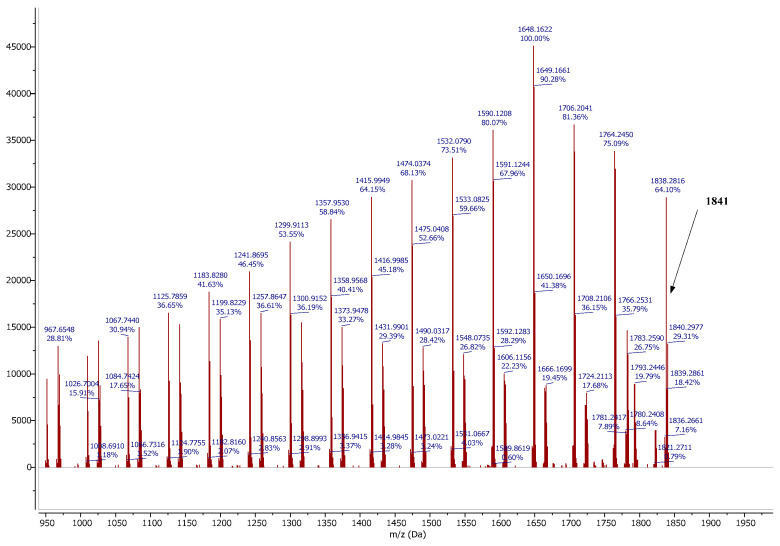
ESI (+)-HRMS/MS spectrum (positive-ion mode) of the oligomers’ parent ion *m*/*z* 1841.

The ^1^H-NMR and ^13^C-NMR provide information about the polymer’s structure, but little information about its molecular mass is available. In this regard, the ESI-HRMS offers additional information about the shape/size selectivity and *m*/*z* [49]. Various techniques can be employed to analyze low-molecular-weight polymers, providing a lot of information about the polymer’s structure [49,50]. In this study, the sample was prepared by dissolving a small quantity in chloroform and via simple methanol dilution, followed by direct injection into the ESI source at a rate of 5 mL/min. The spectrum shows the mass of each polymer formed, as well as its degree of polymerization. In Figure 7, *m*/*z* 621.41778 can be attributed to the n = 7 (M + H) polymer. The signals belonging to the main series occur in the spectrum, with a mass difference of 58 Da between the *m*/*z* values, which can be attributed to the loss of a neutral molecule, namely 2-propanone, from the end of the polymer chain [47]. The ESI-HRMS spectrum of the obtained oligomer is presented in Figure 8. Due to their varying degrees of oligomerization and composition, single-charged ions are grouped in multiple clusters in the spectrum.

The ESI-HRMS/MS spectrum shown in Figure 8 was attained from the precursor ions at *m*/*z* 1841, leading to the formation of two series of product ions. The first main series of ions (*m*/*z* 1764, 1706, 1648, 1590, etc.) corresponds to the PHB oligomers (Figure 7), while the second set of product ions (1782, 1724, 1666, 1608, etc.) corresponds to another fragmentation pattern; in this case, the mass difference is also 58 Da. The functionalization and end groups of PHB polymer were analyzed using the ESI-MS technique. The presence of carboxylic and unsaturated groups was not confirmed via NMR analysis.

#### 3.6.3. Carbon Nuclear Magnetic Resonance (^13^C-NMR)

**Figure 9:**
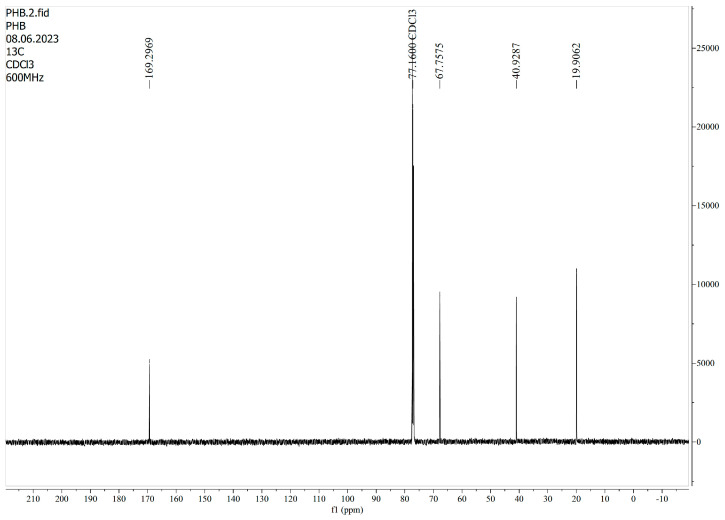
^13^C-NMR spectrum of the obtained PHB.

Figure 9 shows the ^13^C-NMR spectrum of the obtained PHB with the following signals: 169.9 (CO), 67.8 (-CH-), 40.9 (CH_2_), and 19.9 (CH_3_) ppm. The signal at 77 ppm was attributed to solvent. The ^13^C-NMR results are in agreement with those obtained by Shaw et al. (1994) [51]. 

#### 3.6.4. FT-IR Spectrum

**Figure 10:**
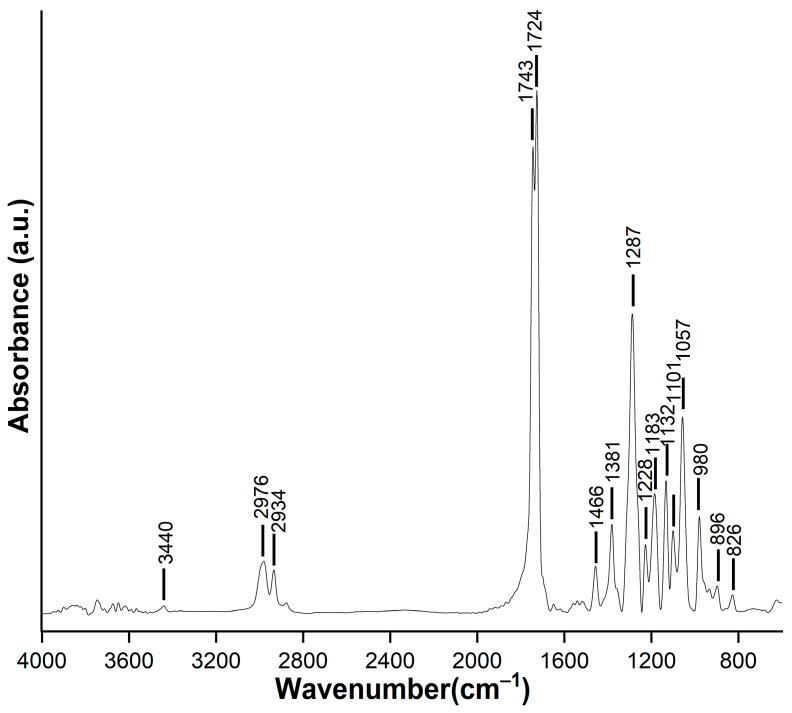
FT-IR spectrum of PHB.

The FT-IR spectrum of the PHB presented in Figure 10 displays the presence the characteristic peaks: two intense peaks at 2976 and 2934 cm^−1^ corresponding to the C-H stretching of -CH_3_ and -CH_2_ groups; three intense peaks at 1743, 1724, and 1287 cm^−1^ assigned to the ester carbonyl groups C=O and asymmetric O-C stretching vibrations; and three peaks at 1381, 1466, and 3440 cm^−1^ attributed to the -CH_3_, -CH_2_, -CH, and O-H groups. The peaks from 1132 cm^−1^ to 826 cm^−1^ were associated with the stretching vibrations of C-O and C-C, while that at 1183 cm^−1^ was associated with the asymmetric stretching vibration of the C-O-C group. The peaks at 1300 and 1100 cm^−1^ correspond to the symmetric stretching of the C-O-C group, according to Heitmann et al. (2016) [52], who reported the characterization of a nanostructured niobium oxyhydroxide dispersed PHB film, and Selvaraj et al. (2021) [53].

The band at 839.2 cm^−1^ was assigned to the crystalline phase of PHB, whereas the band at 1056 cm^−1^ indicated the amorphous phase in the sample. The ratio between the C-O (intensity at 1218 cm^−1^) and -CH_2_ (intensity at 1455 cm^−1^) bands was low in PHB, resulting in the low crystallinity of the product. 

#### 3.6.5. TGA/DTG Analysis

**Figure 11:**
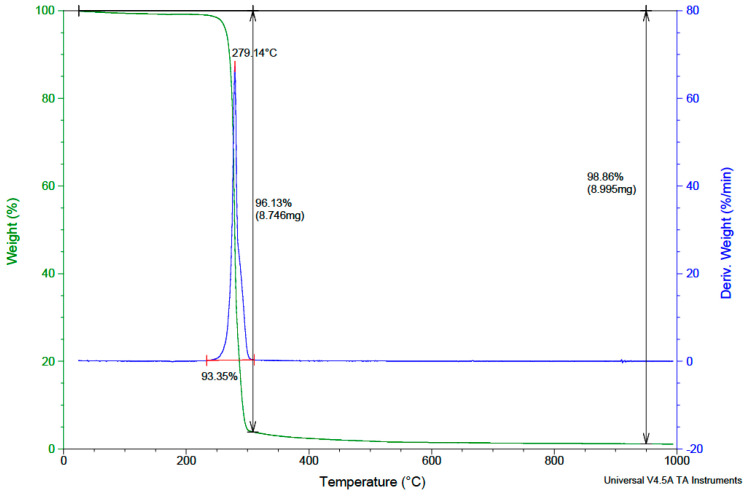
TGA/DTG curves of PHB.

Figure 11 shows the obtained TGA/DTG curves of the PHB. The PHB white powder was heated up to 1000 °C in nitrogen atmosphere at a rate of 10 °C/min. The melting point of PHB appears at a high temperature (279.14 °C) as an isolated peak, indicating that the compound is of high purity. The melting temperature confirms the presence of butyrate units, with no additional residues being detected. The total mass loss was 98.86%, which highlighted the high purity of the PHB. A TGA was conducted to observe the thermal stability of the synthesized PHB. The polymer was degraded completely at 300 °C. The same results were obtained for PHB derived from whey, as reported by Israni et al. (2020) [22]. 

#### 3.6.6. XRD Analysis 

**Figure 12:**
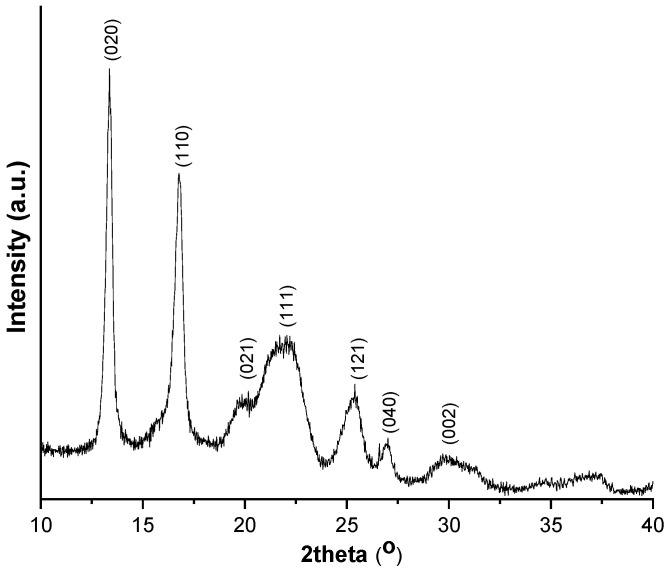
XRD pattern of the PHB sample.

The XRD pattern of the PHB sample presented in Figure 12 shows six intense peaks centered at 2θ = 13.37, 16.79, 19.63, 22.21, 25.60, 27.01, and 30.03°, attributed to the (020), (110), (021), (111), (121), (040), and (002) planes, respectively, indicating an orthorhombic unit cell [54,55]. The XRD patterns are almost identical to the PHB synthetized microbial fermentation by *Bacillus megaterium* and *Cupriavidus necator*: the peaks at around 22° (2θ) indicate the presence of a significant PHB fraction in a crystalline state, while those at around 25° and 27° (2θ) confirmed the partial crystalline nature of PHB [52]. The crystallinity degree of PHB (43.1%) produced by *Bacillus megaterium* ATCC 14581 is comparable to those reported for PHB obtained using *Bacillus megaterium* (MTCC 453) [56]. Previous studies suggested that the polymer matrix has a helical conformation with two anti-parallel chains in the rhombic unit cell within a crystalline unit cell [57].

## 4. Conclusions

In this study, a microwave irradiation pretreatment was used for cellulose and lignin separation, delignification was performed with ammonia for cellulose separation, and enzymatic hydrolysis was performed using *Trichoderma reesei* ATCC 26921 and β-glucosidase enzymes. The fermentation of soluble sugars to PHB was carried out using the *Bacillus megaterium* ATCC 14581 strain. The optimized microwave pretreatment conditions were determined using response surface methodology. The enzymatic hydrolysis yield of the delignified biomass gives the highest sugar content compared to pretreated biomass. PHB was produced from lignocellulosic biomass through various steps, namely pretreatment, delignification, enzymatic hydrolysis, and fermentation. The current study provides a sustainable technology for the conversion of wastes into high-value-added chemicals. The production of PHB from lignocellulosic wastes has the advantage of decreasing the final production cost. Further studies will be focused on reducing the production steps by removing delignification and combining enzymatic hydrolysis and fermentation in the SSF process in order to reduce costs and improve the scalability of the PHB.

## Figures and Tables

**Table 1:** Central composite design of the lignocellulosic biomass pretreatment.

Input Variables	Symbols	Levels		
		Low Factorial (−1)	Center Point (0)	High Factorial (+1)
Temperature (°C)	X_1_	180	200	220
Time (min)	X_2_	10	20	30

**Table 2:** Chemical compositions of pretreated biomass at various temperatures and reaction times (data represent mean ± standard deviation, *n* = 3).

Temperature (°C)	180	180	180	200	200	200	220	220	220
Time (min)	10	20	30	10	20	30	10	20	30
Solid yield (% of raw material, dry biomass)	75.2 ± 2.1	68.3 ± 1.8	64.2 ± 1.5	70.5 ± 2.3	62.1 ± 3.2	56.3 ± 2.1	58.2 ± 2.6	50.1 ± 2.1	42.3 ± 1.8
Cellulose (% of autohydrolyzed biomass, dry biomass)	38.4 ± 1.1	40.2 ± 1.5	42.3 ± 1.4	35.4 ± 1.6	37.5 ± 1.8	43.2 ± 2.0	42.2 ± 2.1	46.2 ± 1.5	41.2 ± 1.6
Hemicelluloses (% of autohydrolyzed biomass, dry biomass)	20.1 ± 1.1	18.2 ± 1.1	14.3 ± 1.2	17.3 ± 0.9	10.1 ± 0.8	7.6 ± 0.06	4.2 ± 0.02	2.1 ± 0.01	1.8 ± 0.02
Lignin (% of autohydrolyzed biomass, dry biomass)	37.2 ± 1.3	38.6 ± 1.2	41.6 ± 1.6	44.6 ± 1.4	48.2 ± 1.2	46.8 ± 1.5	46 ± 1.8	45.6 ± 2.1	42.2 ± 1.7
Solid compositions (% of autohydrolyzed biomass, dry biomass)	95.7 ± 2.1	97 ± 3.2	98.2 ± 3.1	97.3 ± 2.5	95.8 ± 2.4	97.6 ± 2.8	90.4 ± 1.7	91.8 ± 1.9	97.6 ± 2.4

**Table 3:** Summary for the RSM model.

	s	R^2^ (%)	R^2^ (adj) (%)	R^2^ (Predicted) (%)
Solid yield	1.31	99.20	98.40	95.95
Cellulose	1.09	94.68	93.50	91.29
Hemicelluloses	1.84	95.30	92.94	90.68
Lignin	1.34	95.71	91.41	91.25

**Table 4:** Analysis of variance for solid yield.

Source	DF	Adj SS	Adj MS	F-Value	*p*-Value
Model	5	1980.73	396.15	10.40	<0.0001
Linear	2	1917.99	959.00	25.18	<0.0001
X_1_	1	1073.39	1073.39	28.18	<0.0001
X_2_	1	844.61	844.61	22.17	<0.0001
Square	2	44.73	22.37	0.59	0.565
X_1_ X_1_	1	17.57	17.57	0.46	0.504
X_2_ X_2_	1	27.16	27.16	0.71	0.408
Two-Way Interaction	1	18.01	18.01	0.47	0.499
X_1_ X_2_	1	18.01	18.01	0.47	0.499
Error	21	799.93	38.09		
Lack-of-Fit	3	180.21	60.07	1.74	0.194
Pure Error	18	619.72	34.43		
Total	26	2780.66			

DF—degree of freedom; SS—sum of squares; MS—mean sum of squares.

**Table 5:** PHB obtained in the current study compared with the results from the literature.

Carbon Source	Pretreatment Used	Microorganism	PHB	Reference
			C (g/L)	
Rice straw	Sulfuric acid pretreatment	*Bacillus megaterium* B-10	1.496	[41]
Lignocellulosic biomass (soybean husk, sunflower husk, wood straw)	Alkaline pretreatment	*Ralstonia eutropha* ATCC 17699	11.42 g/L PHB accumulation 74.45%	[42]
Rapeseed meal hydrolysates	Acid hydrolysis	*Bacillus megaterium* DSM 319	15.0 g/L	[43]
Lignocellulosic substrates (rice straw, corn husk, sugarcane bagasse, newspaper, wheat bran)	Biological pretreatment (*Pleurotus ostreatus* MTCC 142)	*Bacillus megaterium Ti3*	1 g/L (57.8% after 48 h)	[44]
Marine environment	-	*Bacillus megaterium*	5.61 g/L	[45]
Sugar beet molasses		*Bacillus megateriumuyuni* S29	60 g P(3HB)/100 g cell dry biomass	[46]
Cherry orchard waste	Microwave irradiation and ammonia delignification	*Bacillus megaterium* ATCC 14581	3.02 g/L	This study

## Data Availability

Data are contained within the article.

## Supplementary Materials

The following supporting information can be downloaded at: https://www.mdpi.com/article/10.3390/polym15234488/s1.

## References

[1] Thomas A.P., Kasa V.P., Dubey B.K., Sen R., Sarmah A.K. (2023). Synthesis and commercialization of bioplastics: Organic waste as a sustainable feedstock. Sci. Total Environ..

[2] Stančin H., Strezov V., Mikulčić H. (2023). Life cycle assessment of alternative fuel production by co-pyrolysis of waste biomass and plastics. J. Clean. Prod..

[3] Zhou W., Bergsma S., Colpa D.I., Euverink G.-J.W., Krooneman J. (2023). Polyhydroxyalkanoates (PHAs) synthesis and degradation by microbes and applications towards a circular economy. J. Environ. Manag..

[4] Mehrpouya M., Vahabi H., Barletta M., Laheurte P., Langlois V. (2021). Additive manufacturing of polyhydroxyalkanoates (PHAs) biopolymers: Materials, printing techniques, and applications. Mater. Sci. Eng..

[5] Noveli L.D.D., Cayavedra S.M., Rene E.R. (2021). Polyhydroxyalkanoate (PHA) production via resource recovery from industrial waste streams: A review of techniques and perspectives. Bioresour. Technol..

[6] Westlie A.H., Quinn E.C., Parker C.R., Chen E.Y.-X. (2022). Synthetic biodegradable polyhydroxyalkanoates (PHAs): Recent advances and future challenges. Prog. Polym. Sci..

[7] Muiruri J.K., Yeo J.C.C., Soo X.Y.D., Wang S., Liu H., Kong J., Cao J., Tan B.H., Suwardi A., Li Z. (2023). Recent advances of sustainable Short-chain length polyhydroxyalkanoates (Scl-PHAs)—Plant biomass composites. Eur. Polym. J..

[8] Sohn Y.J., Son J., Lim H.J., Lim S.H., Park S.J. (2022). Valorization of lignocellulosic biomass for polyhydroxyalkanoate production: Status and perspectives. Bioresour. Technol..

[9] González-Rojo S., Díez-Antolínez R. (2023). Production of polyhydroxyalkanoates as a feasible alternative for an integrated multiproduct lignocellulosic biorefinery. Bioresour. Technol..

[10] Steinbüchel A., Valentin H.E. (1995). Diversity of bacterial polyhydroxyalkanoic acids. FEMS Microbiol. Lett..

[11] Yan X., Li D., Ma X., Li J. (2021). Bioconversion of renewable lignocellulosic biomass into multicomponent substrate via pressurized hot water pretreatment for bioplastic polyhydroxyalkanoates accumulation. Bioresour. Technol..

[12] Silva L.F., Taciro M.K., Ramos M.E.M., Carter J.M., Pradella J.G.C., Gomez J.G.C. (2004). Poly-3-hydroxybutyrate (P3HB) production by bacteria from xylose, glucose and sugarcane bagasse hydrolysate. J. Ind. Microbiol. Biotechnol..

[13] Bondar M., Pedro F., Oliveira M.C., da Fonseca M.M.R., Cesário M.T. (2022). Red algae industrial residues as a sustainable carbon platform for the co-production of poly-3-hydroxybutyrate and gluconic acid by *Halomonas boliviensis*. Front. Bioeng. Biotechnol..

[14] Martínez-Herrera R.E., Alemán-Huerta M.E., Rutiaga-Quiñones O.M., de Luna-Santillana E.J., Elufisan T.O. (2023). A comprehensive view of *Bacillus cereus* as a polyhydroxyalkanoate (PHA) producer: A promising alternative to Petroplastics. Process Biochem..

[15] Khomlaem C., Aloui H., Singhvi M., Kim B.S. (2023). Production of polyhydroxyalkanoates and astaxanthin from lignocellulosic biomass in high cell density membrane bioreactor. Chem. Eng. J..

[16] Nygaard D., Yashchuk O., Noseda D.G., Araoz B., Hermida E.B. (2021). Improved fermentation strategies in a bioreactor for enhancing poly(3-hydroxybutyrate) (PHB) production by wild type *Cupriavidus necator* from fructose. Helion.

[17] Huang Z., Liang B., Wang F., Ji Y., Gu P., Fan X., Li Q. (2023). Response surface optimization of poly-β-hydroxybutyrate synthesized by *Bacillus cereus* L17 using acetic acid as carbon source. Int. J. Biol. Macromol..

[18] Kim B., Oh S.J., Hwang J.H., Kim H.J., Shin N., Bhatia S.K., Jeon J.-M., Yoon J.-J., Yoo J., Ahn J. (2023). Polyhydroxybutyrate production from crude glycerol using a highly robust bacterial strain *Halomonas* sp. YLGW01. Int. J. Biol. Macromol..

[19] Cristea A., Baricz A., Leopold N., Floare C.G., Borodi G., Tripon S., Bulzu P.A., Andrei A.-S., Cadar O., Levei E.A. (2018). Polyhydroxybutyrate production by an extremely halotolerant *Halomonas elongate* strain isolated from the hypersaline meromictic Fara Fund Lake. J. Appl. Microbiol..

[20] Chiou C.-Y., Wang H.-H., Shaw G.-C. (2002). Identification and characterization of the non-PTS fru locus of *Bacillus megaterium* ATCC 14581. Mol. Genet. Genom..

[21] Cal A.J., Kibblewhite R.E., Sikkema W.D., Torres L.F., Hart-Cooper W.M., Orts W.J., Lee C.C. (2021). Production of polyhydroxyalkanoate copolymers containing 4-hydroxybutyrate in engineered *Bacillus megaterium*. Int. J. Biol. Macromol..

[22] Israni N., Venkatachalam P., Gajaraj B., Nadumane K., Varalakshmi K.N., Shivakumar S. (2020). Whey valorization for sustainable polyhydroxyalkanoate production by *Bacillus megaterium*: Production, characterization and in vitro biocompatibility evaluation. J. Environ. Manag..

[23] Senila L., Cadar O., Kovacs E., Gal E., Dan M., Stupar Z., Simedru D., Senila M., Roman C. (2023). L-Poly(Lactic Acid) Production by Microwave Irradiation of Lactic Acid Obtained from Lignocellulosic Wastes. Int. J. Mol. Sci..

[24] Gunalan S., Thangaiag A., Rathnasamy V.K., Janaki J.G., Thiyagarajan A., Kuppusamy S., Arunachalam L. (2023). Microwave-assisted extraction of biomolecules from moringa (*Moringa oleifera* Lam.) leaves var. PKM 1: A optimization study by response surface methodology (RSM). Kuwait J. Sci..

[25] Sharma L., Alam N.M., Roy S., Satya P., Kar G., Ghosh S., Goswami T., Majumadar B. (2023). Optimization of alkali pretreatment and enzymatic saccharification of jute (*Corchorus olitorius* L.) biomass using response surface methodology. Bioresour. Technol..

[26] Thuoc D.V., Chung N.T., Hatti-Kaul R. (2021). Polyhydroxyalkanoate production from rice straw hydrolysate obtained by alkaline pretreatment and enzymatic hydrolysis using *Bacillus* strains isolated from decomposing straw. Bioresour. Bioprocess..

[27] Miller G.L. (1959). Use of dinitrosalicylic acid reagent for determination of reducing sugar. Anal. Chem..

[28] Dietrich K., Oliveira-Filho E.R., Dumont M.-J., Gomez J.G.C., Taciro M.K., da Silva L.F., Orsat V., Rio L.F.D. (2020). Increasing PHB production with an industrially scalable hardwood hydrolysate as a carbon source. Ind. Crops Prod..

[29] Kucera D., Pernicová I., Kovalcik A., Mullerova L., Sedlacek P., Mravec F., Nebesarova J., Kalina M., Marova I., Krzyzanek V. (2018). Characterization of the promising poly(3-hydroxybutyrate) producing halophilic bacterium *Halomonas halophila*. Bioresour. Technol..

[30] Hathi Z.J., Haque M.A., Priya A., Qin Z.-H., Huang S., Lam C.H., Ladakis D., Pateraki C., Mettu S., Koutinas A. (2022). Fermentative bioconversion of food waste into biopolymer poly(3-hydroxybutyrate-*co*-3-hydroxyvalerate) using *Cupriavidus necator*. Environ. Res..

[31] Senila L., Kovacs E., Scurtu D.A., Cadra O., Becze A., Senila m., Levei E.A., Dumitras D.A., Tenu I., Roman C. (2020). Bioethanol Production from Vineyard Waste by Autohydrolysis Pretreatment and Chlorite Delignification via Simultaneous Saccharification and Fermentation. Molecules.

[32] Bhati R., Samantaray S., Sharma L., Mallick N. (2010). Poly-β-hydroxybutyrate accumulation in cyanobacteria under photoautotrophy. Biotechnool. J..

[33] Rabiej M. (2003). Application of the genetic algorithms and multi-objective optimization to the resolution of X-ray diffraction curves of semicrystalline polymers. Fibers Text. East. Eur..

[34] Shangdiar S., Cheng P.-C., Chen S.-C., Amesho K.T.T., Ponnusamy V.K., Lin Y.-C. (2023). Enhancing sugar yield for bioconversion of rice straw: Optimization of Microwave-assisted Pretreatment using dilute acid hydrolysis. Environ. Technol. Innov..

[35] Sipponen M.H., Osterberg M. (2019). Aqueous Ammonia Pre-treatment of Wheat Straw: Process Optimization and Broad Spectrum Dye Adsorption on Nitrogen-Containing Lignin. Front. Chem..

[36] Tsafrakidou P., Moutsoglou A., Prodromidis P., Moschakis T., Goula A., Biliaderis C.G., Michaelidou A.-M. (2023). Aqueous ammonia soaking pretreatment of spent coffee grounds for enhanced enzymatic hydrolysis: A bacterial cellulose production application. Sustain. Chem. Pharm..

[37] Park Y.C., Kim J.S. (2012). Comparison of various alkaline pretreatment methods of lignocellulosic biomass. Energy.

[38] Kristensen J.B., Felby C., Jørgensen H. (2009). Yield-determining factors in high-solids enzymatic hydrolysis of lignocellulose. Biotechnol. Biofuels.

[39] Saratale R.G., Cho S.-K., Kadam A.A., Ghodake G.S., Kumar M., Bharagava R.N., Varjani S., Nair S., Kim D.-S., Shin H.-S. (2022). Developing Microbial Co-Culture System for Enhanced Polyhydroxyalkanoates (PHA) Production Using Acid Pretreated Lignocellulosic Biomass. Polymers.

[40] Yamane T. (1992). Yield of Poly-3-Hydroxybutyrate from Various Carbon Sources: A Theoretical Study. Biotechnol. Bioeng..

[41] Li J., Yang Z., Zhang K., Liu M., Liu D., Yan X., Si M., Shi Y. (2021). Valorizing waste liquor from dilute acid pretreatment of lignocellulosic biomass by *Bacillus megaterium* B-10. Ind. Crops Prod..

[42] Saratale G.D., Oh M.-K. (2015). Characterization of poly-3-hydroxybutyrate (PHB) produced from *Ralstonia eutropha* using an alkali-pretreated biomass feedstock. Int. J. Biol. Macromol..

[43] Tadi S.R.R., Ravindran S.D., Balakrishnan R., Sivaprakasam S. (2021). Recombinant production of poly-(3-hydroxybutyrate) by *Bacillus megaterium* utilizing millet bran and rapeseed meal hydrolysates. Bioresour. Technol..

[44] De Sousa L., Manasa Y., Shivakumar S. (2020). Bioconversion of lignocellulosic substrates for the production of polyhydroxyalkanoates. Biocatal. Agric. Biotechnol..

[45] Mohanrasu K., Rao R.G.R., Dinesh G.H., Zhang K., Prakash G.S., Song D.-P., Muniyasamy S., Pugazhendhi A., Jeyakanthan J., Arun A. (2020). Optimization of media components and culture conditions for polyhydroxyalkanoates production by *Bacillus megaterium*. Fuel.

[46] Schmid M.T., Song H., Raschbauer M., Emerstorfer F., Omann M., Stelzer F., Neureiter M. (2019). Utilization of desugarized sugar beet molasses for the production of poly(3-hydroxybutyrate) by halophilic *Bacillus megaterium* uyuni S29. Process Biochem..

[47] Nishida M., Tanaka T., Hayakawa Y., Nishida M. (2018). Solid-State Nuclear Magnetic Resonance (NMR) and Nuclear Magnetic Relaxation Time Analyses of Molecular Mobility and Compatibility of Plasticized Polyhydroxyalkanoates (PHA) Copolymers. Polymers.

[48] Thammasittirong A., Saechow S., Thammasittirong S.N. (2017). Efficient polyhydroxy butyrate production from *Bacillus thuringiensis* using sugarcane juice substrate. Turk. J. Biol..

[49] Rizzarelli P., Rapisarda M. (2023). Matrix-Assisted Laser Desorption and Electrospray Ionization Tandem Mass Spectrometry of Microbial and Synthetic Biodegradable Polymers. Polymers.

[50] Ekere I., Johnston B., Tchuenbou-Magaia F., Townrow D., Wojciechowski S., Marek A., Zawadiak J., Zieba M., Sikorska W., Adamus G. (2022). Bioconversion Process of Polyethylene from Waste Tetra Pak^®^ Packaging to Polyhydroxyalkanoates. Polymers.

[51] Shaw G.L., Melby M.K., Horowitz D.M., Keeler J., Sanders J.K.M. (1994). Nuclear magnetic resonance relaxation studies of poly(hydroxybutyrate) in whole cells and in artificial granules. Int. J. Biol. Macromol..

[52] Heitmann A.P., Patrício P.S.O., Coura I.R., Pedroso E.F., Souza P.P., Mansur H.S., Mansur A., Oliveira L.C.A. (2016). Nanostructured niobium oxyhydroxide dispersed Poly(3-hydroxybutyrate) (PHB) films: Highly efficient photocatalysts for degradation methylene blue dye. Appl. Catal. B Environ..

[53] Selvaraj K., Vishvanathan N., Dhandapani R. (2021). Screening, optimization and characterization of poly hydroxy butyrate from fresh water microalgal isolates. Int. J. Biobased Plast..

[54] Mottin A.C., Ayres E., Oréfice R.L., Câmara J.J.D. (2016). What changes in poly (3-hydroxybutyrate) (PHB) when processed as electrospun nanofibers or thermo-compression molded film?. Mater. Res..

[55] Pradhan S., Dikshit P.K., Moholkar V.S. (2018). Production, ultrasonic extraction, and characterization of poly (3-hydroxybutyrate) (PHB) using *Bacillus megaterium* and *Cupriavidus necator*. Polym. Advan. Technol..

[56] Skrbić Z., Divjaković V. (1996). Temperature influence on changes of parameters of the unit cell of biopolymer PHB. Polymer.

[57] Siracusa V., Karpova S., Olkhov A., Zhulkina A., Kosenko R., Iordanskii A. (2020). Gas Transport Phenomena and Polymer Dynamics in PHB/PLA Blend Films as Potential Packaging Materials. Polymer.

