# Supplementary material for: Poly(3-hydroxybutyrate) Production from Lignocellulosic Wastes Using Bacillus megaterium ATCC 14581
Source: Polymers (Basel). 2023 Nov 22;15(23):4488. doi: 10.3390/polym15234488 (PMC10708134; doi:10.3390/polym15234488)
Supplement: Supplementary file 1 [file polymers-15-04488-s001.zip › polymers-2674135-supplementary.pdf]

## Supplementary Material

# Poly(3-hydroxybutyrate) production from lignocellulosic wastes using *Bacillus megaterium* ATCC 14581

**Lacrimioara Senila [1,*], Emese Gál [2], Eniko Kovacs [1,3], Oana Cadar[1], Monica Dan[4], Marin Senila[1], Cecilia Roman[1]**

**Table S1.** Analysis of variance for cellulose

| Source | DF | Adj SS | Adj MS | F-Value | P-Value |
|---|---|---|---|---|---|
| Model | 5 | 290.641 | 58.128 | 3.57 | 0.[illegible] |
| Linear | 2 | 84.125 | 42.063 | 2.58 | 0.099 |
| $X_1$ | 1 | 15.680 | 15.680 | 0.96 | 0.338 |
| $X_2$ | 1 | 68.445 | 68.445 | 4.20 | 0.053 |
| Square | 2 | 206.508 | 103.254 | 6.34 | 0.007 |
| $X_1 X_1$ | 1 | 206.507 | 206.507 | 12.68 | 0.002 |
| $X_2 X_2$ | 1 | 0.002 | 0.002 | 0.00 | 0.992 |
| 2-Way Interaction | 1 | 0.008 | 0.008 | 0.00 | 0.983 |
| $X_1 X2$ | 1 | 0.008 | 0.008 | 0.00 | 0.983 |
| Error | 21 | 342.054 | 16.288 | | |
| Lack-of-Fit | 3 | 52.566 | 17.522 | 1.09 | 0.379 |
| Pure Error | 18 | 289.489 | 16.083 | | |
| Total | 26 | 632.695 | | | |

**Table S2**. Analysis of variance for hemicelluloses

| Source | DF | Adj SS | Adj MS | F-Value | P-Value |
|---|---|---|---|---|---|
| Model | 5 | 1176.92 | 235.385 | 69.42 | <0.0001 |
| Linear | 2 | 1150.51 | 575.254 | 169.66 | <0.0001 |
| $X_1$ | 1 | 990.42 | 990.422 | 292.11 | <0.0001 |
| $X_2$ | 1 | 160.09 | 160.086 | 47.21 | <0.0001 |
| Square | 2 | 17.78 | 8.890 | 2.62 | 0.096 |
| $X_1 X_1$ | 1 | 14.39 | 14.394 | 4.25 | 0.052 |
| $X_2 X_2$ | 1 | 3.39 | 3.385 | 1.00 | 0.329 |
| 2-Way Interaction | 1 | 8.64 | 8.636 | 2.55 | 0.125 |
| $X_1 X2$ | 1 | 8.64 | 8.636 | 2.55 | 0.125 |
| Error | 21 | 71.20 | 3.391 | | |
| Lack-of-Fit | 3 | 42.66 | 14.221 | 8.97 | 0.001 |

| | | | | | |
|---|---|---|---|---|---|
| Pure Error | 18 | 28.54 | 1.586 | | |
| Total | 26 | 1248.13 | | | |

**Table S3**. Analysis of variance for lignin

| **Source** | **DF** | **Adj SS** | **Adj MS** | **F-Value** | **P-Value** |
|---|---|---|---|---|---|
| Model | 5 | 342.548 | 68.510 | 38.40 | 0.000 |
| Linear | 2 | 161.575 | 80.788 | 45.29 | 0.000 |
| $X_1$ | 1 | 159.907 | 159.907 | 89.64 | 0.000 |
| $X_2$ | 1 | 1.668 | 1.668 | 0.94 | 0.345 |
| Square | 2 | 138.032 | 69.016 | 38.69 | 0.000 |
| $X_1 X_1$ | 1 | 128.591 | 128.591 | 72.08 | 0.000 |
| $X_2$ $X_2$ | 1 | 9.442 | 9.442 | 5.29 | 0.032 |
| 2-Way Interaction | 1 | 42.941 | 42.941 | 24.07 | 0.000 |
| $X_1$ X2 | 1 | 42.941 | 42.941 | 24.07 | 0.000 |
| Error | 21 | 37.463 | 1.784 | | |
| Lack-of-Fit | 3 | 18.581 | 6.194 | 5.90 | 0.005 |
| Pure Error | 18 | 18.882 | 1.049 | | |
| Total | 26 | 380.011 | | | |
